# Effects of HLEC on the secreted proteins of epithelial ovarian cancer cells prone to metastasize to lymph nodes

**DOI:** 10.7497/j.issn.2095-3941.2013.04.006

**Published:** 2013-12

**Authors:** Xin-Ying Zhang, Fu-Qiang Yin, Li Liu, Ting Gao, He-Yun Ruan, Xiao Guan, Ying-Xin Lu, Dan-Rong Li

**Affiliations:** 1 The Cancer Hospital Affiliated to Guangxi Medical University, Nanning 530021, China;; 2 The Experiment Center of The Guangxi Medical University, Nanning 530021, China

**Keywords:** Ovarian cancer, tumor microenvironment, lymphatic metastasis, human lymphatic capillary endothelial cells, secreted proteins

## Abstract

**Objective:**

To study explores the effect of HLEC on the secreted proteins of epithelial ovarian cancer (EOC) cells (SKOV3-PM4) with directional highly lymphatic metastasis.

**Methods:**

Supernatants of four groups of cultured cells, namely, SKOV3 (A), SKOV3+HLEC (B), SKOV3-PM4 (C), SKOV3-PM4+HLEC (D), were collected, and their proteins were detected by antibody arrays and iTRAQ-2D-LC-MALDI-TOF/TOF/MS. Significantly differential proteins were further analyzed via bioinformatics and validated in human serums and cell media via ELISA.

**Results:**

Results of antibody arrays and mass spectrometry demonstrated that GRN and VEGFA were upregulated in group C (compared with group A), whereas IGFBP7 and SPARC were downregulated in group D (compared with group C). Comprehensive bioinformatics analysis results showed that IGFBP7 and VEGFA were closely linked to each other. Further validation with serums showed statistical significance in VEGFA and IGFBP7 levels among groups of patients with ovarian cancers, benign tumors, and control groups. Two proteins were upegulated in the first group. VEGFA in the control group was downregulated. For IGFBP, upregulation in the control group and down-regulation in the first group were also observed.

**Conclusion:**

The HLEC microenvironment is closely associated with directional metastasis to lymph nodes and with differential proteins including cell stromal proteins and adhesion factors. The upregulation of VEGFA and GRN and the downregulation of SPARC and IGFBP7 are closely associated with directional metastasis to lymph nodes in EOC cells.

## Introduction

The delitescent onset of epithelial ovarian cancer (EOC) is possible. Early metastasis occurs easily, in which lymphatic metastasis is the primary route of metastasis. The mortality rate of EOC is the highest among malignant gynecological tumors. Studies have recently indicated that lymphangiogenesis in tumor is closely related to tumor node metastases, which can be promoted by lymphatic proliferation^1^.

In the previous study, sublines of human ovarian papillary serous carcinoma cells (SKOV3) in lymphatic metastasis were cloned in nude mice, wherein the fourth generation of SKOV3 (SKOV3-PM4) cells were screened and established with directional highly lymphatic metastasis potential^2^. In the present study, we conducted experiments on solitary culture and co-cultures of SKOV3, SKOV3-PM4, and human lymphatic capillary endothelial cells (HLEC) to investigate the effect of HLEC on proteins secreted by EOC cells with directional highly lymphatic metastasis (SKOV3-PM4). This study provides a rationale for the therapy of anti-lymphangiogenesis and anti-lymphatic metastasis among patients with EOC.

## Materials and methods

### Study object

SKOV3 cell lines were purchased from the Center of Cell Resources of Shanghai Institutes for Biological Sciences, whereas SKOV3-PM4 cell lines were prepared by this research group^2^. HLEC was purchased from the US Sciencell Co. Sources of serum specimens were obtained from 20 benign ovarian tumor patients and 20 ovarian carcinoma patients with lymph node metastasis. All of the participants were patients with definite pathological diagnosis made by the Cancer Hospital Affiliated to Guangxi Medical University. The patients with ovarian cancer were 32 to 60 years of age, and the median age was 52. They were not surgically and chemo radio therapeutically treated before blood specimen collection. A total of 20 healthy women (normal physical examinees; 38 to 55 years old; median age was 43) were included in the control group. The participants in the control group had no history of infection and medication within four weeks before the study. The institute received approval of the Ethics Committee as well as consent from the patients.

### Primary reagents

The cytokine antibody array was purchased from the US RayBiotech Co, and the ITRAQ kit was from the US ABI company. The ELISA kit was purchased from the American Abcam Co. Special culture medium (ECM) for HLEC were purchased from the US Sciencell Co., HPLC (1,200 series) from Agilent Co., and Nano-LC separation point target system and 5800 MALDI-TOF/TOF protein analyzer were from the American ABI Co.

### Preparation of supernatant from cell culture

Three groups of cells, namely, SKOV3, SKOV-PM4, and HLEC, were digested at the logarithmic growth phase (10^6^ cells). For co-cultures, 5×10^5^ HLEC cells were added to culture flasks containing the same amount of SKOV3 and SKOV3-PM4 cells. After 24 h, the medium was replaced with basal ECM without serum and cytokine. After 48 h, the supernatant from the cell cultures were collected. The supernatants were then refrigerated at –80 °C after dividing them into different groups, namely, SKOV3 (A), SKOV3+HLEC (B), SKOV3-PM4 (C), SKOV3-PM4+HLEC (D).

### Detection of cytokine antibody array

The procedure was conducted according to the RayBio human cytokine 507 antibody array manual. The cell supernatants were labeled with biotin. The array was balanced and then closed. The specimens were cleansed, incubated with fluorescer-streptavidin, and cleansed again. Images were then obtained using a Uniscand1000 scanner. The data were processed with a Scanalyze software. Standard values of individual antibody expression were then calculated. Cytokines with differential expressions were screened according to the following criteria:

Upregulation: the ratio of group C/A and D/C ≥1.33. Downregulation: the ratio of group C/A and D/C ≤0.77.

### Detection with ITRAQ-2D-LC-MALDI-TOF/TOF/MS

Proteolysis was performed using ITRAQ labels (113, 114, 115, and 116, which correspond to the four groups of samples marked A, B, C, and D, respectively). Chromatographic separation and targeting were conducted according to reference^3^ and the operation manual of the device.

Mass spectrometry (MS) and database retrieval: A 5800 Plus MALDI-TOF/TOF mass spectrum analyzer was used in tandem with MS (MS/MS) to perform identification and relative quantitative analysis of polypeptides. ProteinPilot 2.0 was used to perform retrieval and identification of proteins from the MS data against the NCBInr database. Relative quantitative analysis was conducted for proteins at a confidence level of ≥95% by using the peak area integrals of ions reported from *m*/*z* 114, 115, 116, and 117. Results with *P*≤0.05 were chosen for the report based on the ratio of 115^:^113 and 116^:^115. Manual confirmation was not performed for protein polypeptide matches of more than one high confidence level (no less than 99%), whereas manual confirmation was performed to the fragment ions of the MS/MS spectrum of proteins with matching polypeptide without a high confidence level (no less than 99%).

### Bioinformatics analysis

Bioinformatics analysis was conducted on proteins with intersection of differences in array detection and MS. Proteins with significant differences were chosen for verification in human serum specimens and supernatants from cell cultures via the ELISA method. The instruction manual provided by ABCAM Co. for the ELISA kit was strictly followed.

### Statistical analysis

All data quantification and satistical analyses were performed using SPSS16.0 software. Data were presented as the mean ± SD. Analysis of variance was performed to compare measurement data. *P* value <0.05 were considered statistically significant.

## Results

### Cytokine antibody array detection

By comparing SKOV3-PM4 with SKOV3, 39 differential proteins were found in the SKOV3-PM4 culture supernatant, in which 34 were upregulated and 5 were downregulated. By comparing SKOV3-PM4+HLEC with SKOV3-PM4, 41 differential proteins were found after co-culture, in which 22 were upregulated and 19 were downregulated. The results are shown in **Table 1**.

**Table 1:** Differentially expressed proteins in different cell supernatants

Expression	SKOV3-PM4/SKOV3	SKOV3-PM4+HLEC/SKOV3-PM4
Up-regulation	GRN, CSF2, CCRL2, VEGFA, IFNG, IL17D, FLT3LG, IL2, HBEGF, GH1, CTLA4, IL18, IL4r, IL36RN, IL1, ERBB3, IL8, FGF5, WFIKKN1, EDAR, IL10, FGF6, FGF10, IL2RA, IL9, IL17, FGF4, ICAM5, GDF1, SLPI, CCR7, DEFB1, APLNR, MMP24	ARTN, CD40, SELE, EPO, FGF13 1B, AIMP1, CRIM1, IL15, IL7, IL17, AGRP, CFC1, PROK1, BMP7, GRN, DEFB1, CCR3, TLR3, CSF2, FGF16, VEGFA, ITGAM
Down-regulation	SPARC, IGFBP7, CXCL2, IL37, NRG3	IGFBP7, SPARC, IL2RA, NRG3, IL36RN, IL1, FGF4, CCRL2, IL9, HBEGF, IL18, FGF20, FLT3LG, IL4R, IL10, IL1 R8, ICAM5, MUSK, EDAR

### ITRAQ label combined with MALDI-TOF-TOF MS/MS

After data analysis, SKOV3-PM4 was compared with SKOV3, and 36 differential proteins were present in the SKOV3-PM4 culture supernatant, in which 22 were upregulated and 14 were downregulated.

By comparing SKOV3-PM4+HLEC with SKOV3-PM4, 65 differential proteins were found in the co-culture, in which 37 were upregulated and 28 were downregulated. The expressions of VEGFA and GRN was upregulated, whereas that of SPARC and IGFBP7 was downregulated. This result was consistent with the tendency of the array, as shown in **Table 2**.

**Table 2:** Intersection of differential protein expression in the antibody arrays and iTRAQ-2D-LC-MALDI-TOF/TOF/MS

Project	VEGFA	GRN	SPARC	IGFBP7
SKOV3-PM4/SKOV3 by antibody arrays	1.38	1.33	0.07	0.47
SKOV3-PM4/SKOV3 by ITRAQ-2D-LC-MALDI-TOF/TOF/MS	7.58	3.78	0.57	0.55
SKOV3-PM4+HLEC/SKOV3-PM4 by antibody arrays	3.79	1.46	0.10	0.36
SKOV3-PM4+HLEC/SKOV3-PM4 by ITRAQ-2D-LC-MALDI-TOF/TOF/MS	9.08	4.24	0.05	0.30

### Bioinformatics analysis conducted on differential cytokines

**Figure 1:**
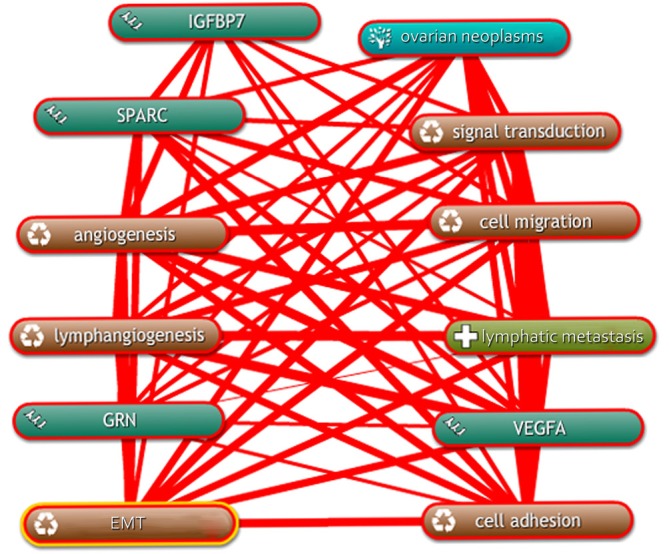
Regulation network of differential proteins and lymphatic metastasis in ovarian cancer.

Bioinformatics analysis was conducted on the screened differential cytokines. The figure of network regulation relationship between the differential proteins and the lymphatic metastasis of EOC was obtained using a Coremine software (**Figure 1**).

**Figure 2:**
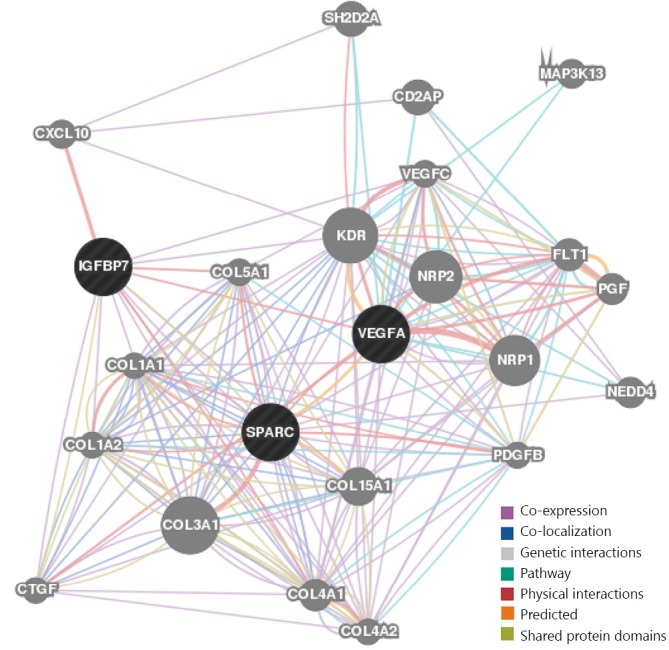
Function prediction of IGFBP7 by GeneMANIA.

**Figure 1** shows different link thicknesses that represent different correlation degrees among proteins, biological processes, and EOC, as well as lymphatic metastasis. Thicker lines represent higher correlations between both ends of the line. The analysis showed that OEC is closely associated with VEGFA, SPARC, and GRN, but it is not correlated with IGFBP7. Interaction network among IGFBP7 and the three proteins that are closely correlated with ovarian cancer were drawn using GeneMania to analyze the relationship between IGFBP7 and lymphatic metastasis of ovarian cancer. In **Figure 2**, the lines in different colors represent different correlations, in which the thickness corresponds to the degree of correlation.

**Figure 2** shows that coexpression and co-localization are apparent among multiple proteins, such as IGFBP7, SPARC, VEGFA and CXCL10. These proteins have common structural domain and interact with one another. In determining the functions of IGFBP7 and other proteins in terms of their biological behaviors, we can speculate that they are possibly important in metastasis. The functions of the relevant proteins were analyzed using a DAVID software. The results show that the proteins are primarily involved in the regulation of cell migration, cell adhesion, and neovascularization (**Table 3**).

**Table 3:** The annotated functions of IGFBP7 and other proteins related to metastasis in GeneMANIA network (as known in **Figure 2**)

GO annotation	Proteins in the network	*P*
Vasculature development	VEGFC, CTGF, VEGFA, COL3A1, COL1A2, COL15A1, COL5A1, KDR	2.20E-10
Angiogenesis	VEGFC, CTGF, VEGFA, COL15A1, KDR	4.30E-6
Positive regulation of cell migration	VEGFC, VEGFA, KDR, CXCL10	4.40E-5
Cell migration	VEGFC, CTGF, COL5A1, KDR	1.20E-3
Cell adhesion	CTGF, IGFBP7, COL3A1, COL15A1, COL5A1	1.80E-3

### ELISA verification

**Figure 3:**
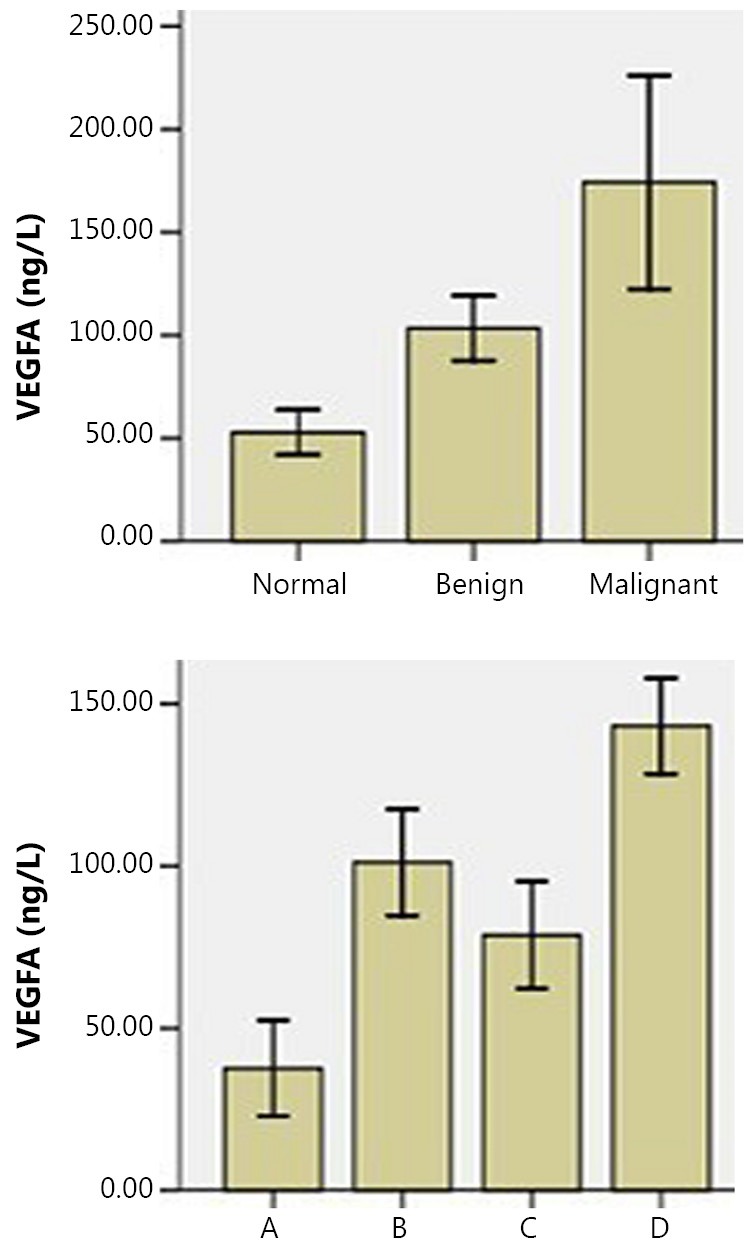
VEGFA concentrations in serum and cell supernatant.

**Figure 4:**
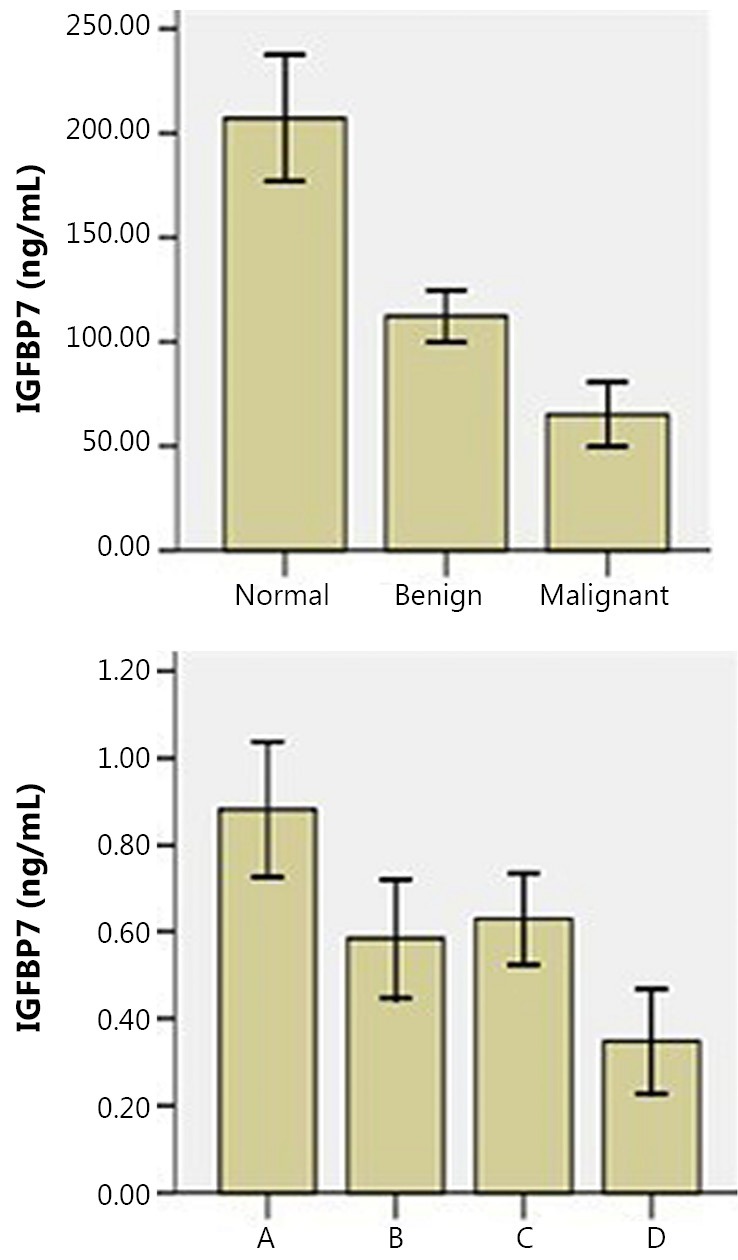
IGFBP7 concentrations in serum and cell supernatant.

Bioinformatic analysis results show that IGFBP7 is closely correlated with VEGFA protein, but no scientific reports on IGFBP7 and ovarian cancer are currently available. Therefore, IGFBP7 and VEGFA were selected for ELISA verification in this study. With regard to VEGFA, any pairwise comparison was statistically significant (*P*<0.05) among the three groups, in which the expression level in the malignant group was the highest. As for IGFBP7, the difference of any pairwise comparison was also statistically significant, in which the tendency was opposite to that of VEGFA, that is, the expression level in the normal group was the highest. In pairwise comparison of the cell supernatants between SKOV3 and SKOV3-PM4 as well as SKOV3-PM4 and SKOV3-PM4+HLEC, the expression tendencies of both proteins were statistically significant (*P*<0.05), which was similar to array detection and MS (**Figures 3****,****4**).

## Discussion

Numerous studies have shown that after tumor cells contact with HLEC in a tumor microenvironment, metastasis and invasion of tumor cells occurred by inducing lymphangiogenesis to drain the lymph fluid and the tumor cells. The new lymphatic vessels provide nutrients and oxygen to promote or maintain the growth of tumor cells. Thus, lymphangiogenesis is an important pathway for invasion and metastasis of tumors^4^. Directional lymphatic metastasis of tumor primarily depends on the migration, adherence, and penetration of tumor cells to HLEC. In the present study, HLEC was used to co-culture with SKOV3-PM4 cells. We simulated the tumor environment of HLEC and used the cytokine antibody array for detection. Bioinformatics analysis showed that the upregulation of VEGFA and GRN, as well as the downregulation of IGFBP7 and SPARC, were correlated with lymphatic metastasis of EOC, and that VEGFA was an important node protein.

It is clear that the angiogenesis factor VEGFA is involved in tumor growth and spread, and promotes angiogenesis by binding VEGFR1 and VEGFR2. VEGFR2 is reported to be expressed in HLEC, and VEGFC and VEGFD can bind VEGFR2 and VEGFR3, mediating lymphangiogenesis^5^. In cursiefen-induced angiogenesis and lymphangiogenesis^6^ in normal cornea without blood and lymph vessels, the function of VEGFA is blocked but that of VEGFC and VEGFD was retained. Angiogenesis and lymphangiogenesis are simultaneously blocked, suggesting that VEGFA simultaneously promotes angiogenesis and lymphangiogenesis. Recently, VEGF-A has been found to function in HLEC by promoting lymphangiogenesis and the lymphatic metastasis of cancer cells. Spannuth^7^ found that the expression level of VEGFR2 in patients with ovarian cancer was high. Qiu^8^ detected the expression of VEGFAmRNA in ovarian cancer by using RT-PCR and immunohistochemistry. The density of the lymph vessels was manually determined, and the results implied that the high expression level of VEGFA is correlated with lymphangiogenesis and lymph node metastasis.

GRN is an autocrine growth factor closely related with the onset and development of various tumors, such as ovarian and esophageal cancer. Liu^9^ tested the expression level of GRN in three types of human ovarian cancer cell lines with different malignant potentialities and demonstrated that the expression level of GRN was closely and positively correlated with tumor cell proliferation and invasiveness. In the classical signal transduction pathway of VEGFA in promoting angiogenesis, GRN is involved in three kinase pathways that regulate tumor growth (MAPK, P13K, and FAK). We speculated that GRN, as an upstream gene protein, might indirectly promote angiogenesis and lymphangiogenesis of tumor by regulating the expression pathway of VEGFA^10^.

SPARC is an extracellular matrix glycoprotein with complex functions and tissue specificity. SPARC in tumor tissues can alter the function of stroma and tumor cells and activate downstream pathways, such as ILK, FAK, and MAPK, especially the expression of relevant factors of angiogenesis, thereby controlling tumor growth and invasion^11^. In an animal model of ovarian cancer^12^, the lack of SPARC upregulated the expression of VEGF, thereby promoting angiogenesis, lymphangiogenesis, and the metastatic ability of cancer cells.

IGFBP7 is a cell adhesion glycoprotein and is widely present in a normal human body. IGFBP7 is relatively abundant in ovaries compared with normal tissues, where its content in corresponding tumor tissues is low^13^. It regulates the growth and adhesion of epithelial cells and stimulates endothelial cells to produce prostacyclin, interact with other cells, and regulate angiogenesis. Various experiments have shown that a tumor-suppressing gene reduces IGFBP7 expression in tumor tissues, such as those in breast cancer. However, no scientific reports on the expression and biological functions of IGFBP7 in ovarian cancer are currently available. The present study showed that the secretion and expression of IGFBP7 in SKOV3-PM4 was lower than those of SKOV3. The expression level of IGFBP7 was even lower after co-culture of SKOV3-PM4 with HLEC, indicating that specific factors secreted by HLEC inhibited the expression of IGFBP7 in tumor cells. The high malignancy and strong lymphatic metastasis ability of the tumor result in a low expression level of IGFBP7. **Figure 2** shows that the structural domain of IGFBP7 is similar to that of SPARC, and IGFBP7 is correlated with multiple proteins, such as CXCL10 and VEGFA. These proteins are involved in biological processes, such as angiogenesis, cell migration, and adhesion, which are closely associated with tumor metastasis. CXCL10 and its receptor CXCR3 are effective inhibitors of angiogenesis, which reduce blood supply to tumor tissues, thereby inhibiting the growth and metastasis of tumors^14^. Inhibition of metastasis by IGFBP7 might be related to its function in adjusting the signal pathway of 1GF-1 and the inhibition of angiogenesis, instead of direct combination with VEGFA^15^.

In summary, this study showed that more than 41 proteins with different expression are present after co-culture of SKOV3-PM4 with HLEC. According to literature, these proteins are involved in functions related to stroma cells, cell adhesion factors, interleukin, chemotactic factor, and cell signal transduction. Further studies on the specific functions and impacts of these proteins on the onset and development of lymph node metastasis of ovarian cancer are warranted.
